# Study on the Binding of Five Plant-Derived Secondary Metabolites to G‑Quadruplexes

**DOI:** 10.1021/acsomega.5c09032

**Published:** 2026-01-05

**Authors:** Lucie Vrtalová, Michaela Dobrovolná, Daniel Platero-Rochart, Aleksandra L. Ptaszek, Václav Brázda, Pedro A. Sánchez-Murcia

**Affiliations:** † Institute of Biophysics of the Czech Academy of Sciences, Královopolská 135, Brno 612 00, Czech Republic; ‡ Brno University of Technology, Faculty of Chemistry, Purkynova 118, Brno 612 00, Czech Republic; § Laboratory of Computer-Aided Molecular Design, Division of Medicinal Chemistry, Otto-Loewi Research Center, 31475 Medical University of Graz, Neue Stiftingtalstrasse 6/III, Graz 8010, Austria; ∥ Christian Doppler Laboratory for High-Content Structural Biology and Biotechnology, Department of Structural and Computational Biology, Max Perutz Laboratories, University of Vienna, Campus Vienna Biocenter 5, Vienna 1030, Austria; ⊥ BioTechmed-Graz, Mozartgasse 12/II, Graz 8010, Austria

## Abstract

Chemical targeting of noncanonical secondary structures of DNA and RNA has emerged as a promising approach for therapeutic development. The most promising targets seem to be four-stranded structures in the G-rich regions of the genome, known as G-quadruplexes (G4s), which are associated with important regulatory regions including promoters. In this study, we tested and modeled the binding of five plant-derived secondary metabolites, known for their antiproliferative activity in vitro, to two G4s found in the human genome: the first at the *c-Myc* proto-oncogene and the second at the human telomere repeat region. Among the tested compounds, brucine exhibited the strongest interaction with both G4 sequences, while ellagic acid demonstrated binding efficacy comparable to that of brucine in the *c-Myc* sequence. Through molecular dynamics simulations and the Markov state model, we explored the binding modes of these ligands, elucidated the G4 stability in the bound state, and investigated the fluorescence quenching effect of thioflavin T (ThT) upon its displacement. The biological effects of these natural compounds were investigated in human cell lines, and the interaction with G4s was verified experimentally using a fluorescence displacement assay and CD spectroscopy. This study demonstrates the interaction of these natural compounds with the G4 structures and their implications for therapeutic targeting.

## Graphical abstract

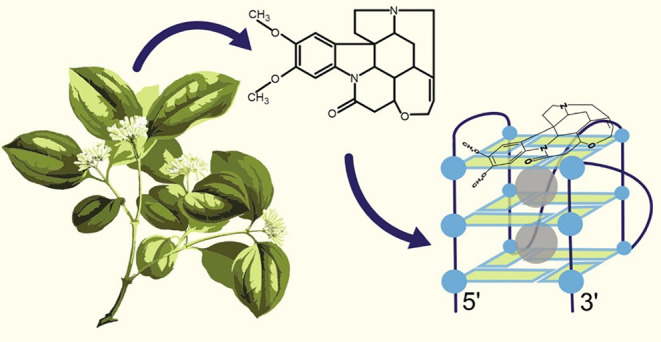

## Introduction

Natural compounds have been cornerstones of medicine for millennia, providing a rich source of therapeutic agents. For centuries, plant-derived secondary metabolites such as brucine,^1,2^ gallic acid,^3^ ellagic acid,^4^ epicatechin,^5^ and epigallocatechin gallate (EGCG)^6,7^ (Figure 1) have been recognized for their diverse pharmacological properties, including potent antioxidant and anticancer activities.^1–4^ All these substances have long-established beneficial effects, particularly their high antioxidant capacity and anticancer activity. The alkaloid brucine has analgesic and anti-inflammatory effects and serves as a muscle relaxant.^8–10^ Gallic acid, present in large quantities in fermented black tea,^11^ plays a role in cardiovascular health by helping to maintain healthy blood pressure levels and offers protection against infections through its antimicrobial properties.^12,13^ Epicatechin and ellagic acid have similar health benefits. It has been shown that they improve cholesterol levels, have a positive effect on the gut microbiome, and their anti-inflammatory effects may be useful in the prevention of inflammatory diseases such as arthritis or in the prevention of neurodegenerative diseases with long-term use.^14–16^ EGCG seems to be beneficial in the prevention of type 2 diabetes due to its ability to regulate the cyclic adenosine monophosphate pathway and protein kinase A, thereby increasing insulin secretion when it is stimulated by glucose.^17,18^ EGCG has also been shown to protect the ends of human telomeres from premature shortening, regulate methylation, and protect skin cells from UV radiation.^19–21^

**Figure 1:**
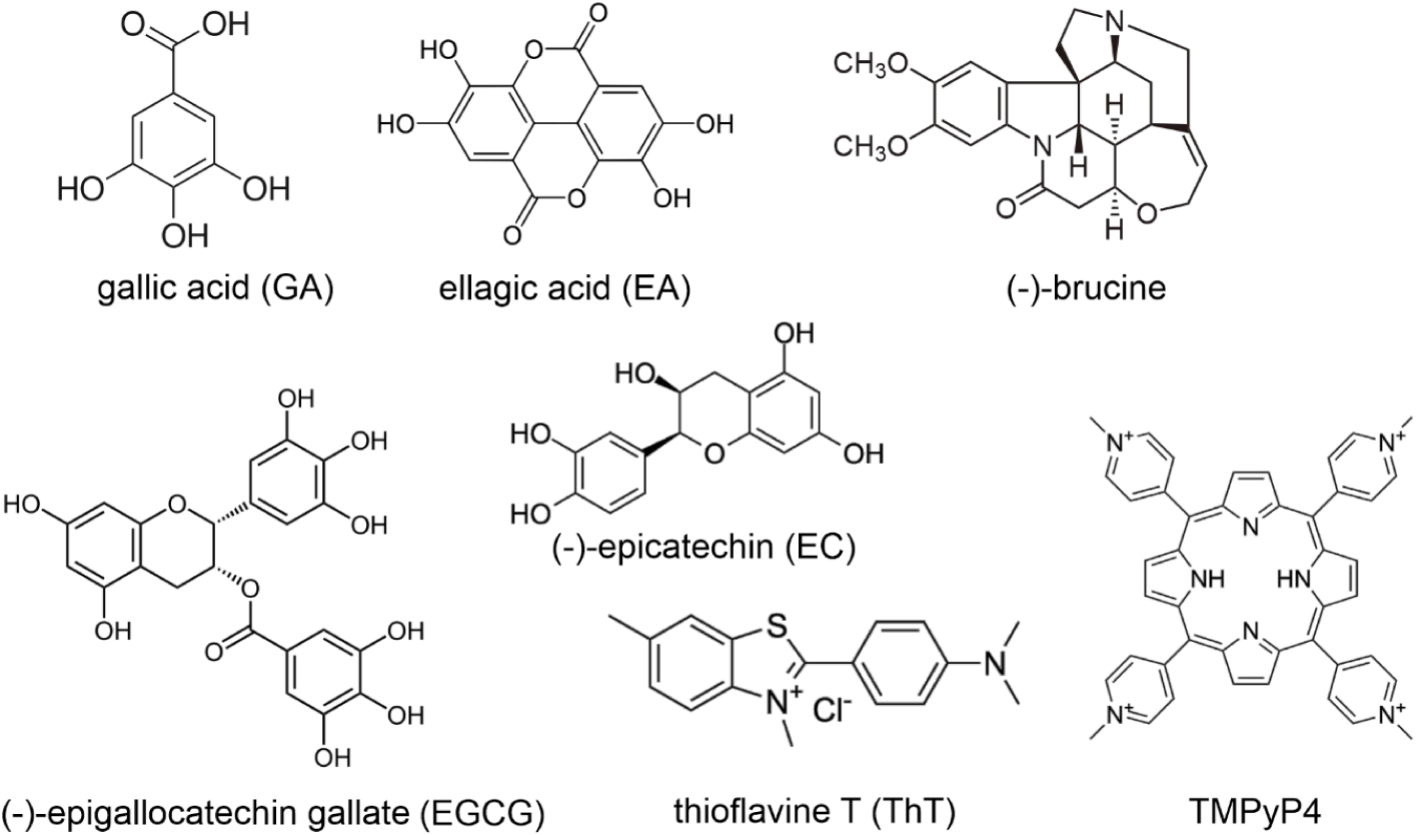
Chemical structures of the tested compounds (no counterions are shown in TMPyP4 for simplicity).^45^

Interestingly, all of these plant-derived compounds have been shown to interact with DNA, especially with G4s. G4s are so-called “noncanonical” DNA structures with a different arrangement from the classic right-handed double helix (B-DNA). In the last decades, it has been demonstrated that these structures are localized in the important regulatory regions of genomes and play a crucial role in many basic biological functions, and they are formed in guanine-rich regions of DNA or RNA. G4s are stabilized by Hoogsteen hydrogen bonding, where four guanine bases associate to form a planar arrangement called a G-tetrad.^22^ Stacked G-tetrads further stabilize the G4 structure through the coordination of monovalent cations such as potassium or sodium^23,24^ and can adopt various topologies.^25,26^ Different G4 structures can form depending on the sequence and length of the guanine-rich regions,^27^ the presence of certain ions,^24^ and the conditions of the cellular environment.^28^ G4 structures have been identified in key regulatory regions of the genome, particularly in telomeres, where they perform a protective function against telomere shortening during each replication,^29^ as well as in oncogene promoters (such as *c-Myc* and *c-Kit*),^30,31^ lysosome-associated genes,^39^ and in various RNA molecules—for example, in untranslated regions (UTRs) of mRNA^32,33^ or in miRNA within small extracellular vesicles.^34^ Thus, the presence and stability of G4s are critical for maintaining appropriate protein levels in the body, and any change in G4 structure or stability can further alter the rate of gene expression.^35–37^

It was shown that brucine binds to G4s in the *c-Myb* promoter region^1^ and that brucine reduces *c-Myb* expression and tumor cell growth; therefore, it is suggested as a potential therapeutic compound in malignant tumors.^8–10^ Gallic acid has been shown to target G4s in rDNA and the *c-Myc* oncogene and to affect gene expression in primary colon cancer cells.^38,39^ Ellagic acid has increased the thermal stability of a G4 structure derived from the promoter of the KRAS proto-oncogene.^40^ Finally, epicatechin derivatives have been observed to bind directly with both single-stranded and double-stranded DNA/RNA.^41^ Although all of these molecules have been shown to interact with G4s and exert interesting biological effects in different cancer models, the molecular aspects of how they bind are missing. It has been demonstrated that molecules that bind and stabilize G4s in oncogene promoters can suppress gene transcription, potentially inhibiting cancer cell growth.^42^ Conversely, destabilizing G4 structures in telomeres may lead to telomere dysfunction and apoptosis in cancer cells.^43^ Therefore, understanding how these natural substances interact with G4s is important for the development of reasonable therapeutics.^44^

Molecular dynamics simulations provide valuable insights into the binding modes, conformational changes, and thermodynamic properties of ligand–G4 complexes, aiding in the design of more potent and selective G4 ligands. This study explored the binding of brucine, gallic acid (GA), ellagic acid (EA), epicatechin (EC), and EGCG to G4s through detailed molecular docking simulations and verified these interactions using biophysical methods. Insights gained from this research could contribute to the development of new therapeutic agents targeting G4s in cancer cells.

## Results and Discussion

### Binding Mode of Thioflavin T to G-Quadruplex

Before exploring the binding of the natural compounds to DNA G4s, we were intrigued to investigate how the fluorescence reporter ThT binds to the *c-Myc* parallel G4. To answer this question, we ran extensive classical molecular dynamics (MD) simulations in the μs-range for the ThT:G4 complex^46,47^ (see Experimental Section). We docked ThT into the DNA using, as a reference, the crystallographic structure of berberine in complex with the *c-Myc* G4 (PDB ID: 7N7D).^48^ ThT is formed by two chemical motifs: a benzothiozole ring and a phenyl ring decorated with a *p*-dimethylamino group. ThT binds to G4 with its sulfur atom oriented toward the potassium ion, the dimethylamino group pointing to the DNA backbone, and the two rings establishing π-stacking interactions with two guanine nucleobases (Figure 2A). Further, ThT interacts alternately with G8 and G13 or with G13 and G4 (Figure 2B). This indicates certain flexibility along the MD simulation, but the root-mean-square deviation (RMSD) for the heavy atoms of ThT along the MD simulation does not exceed 1.3 Å, which indicates no significant conformational change (Figure 2C). We also measured the dihedral angle defined by the atoms S–C6–C12–C17 around the covalent bond connecting both rings in ThT (Figure 2C). The breakage of the system of conjugated π-bonds due to the rotation of the benzothiazole with respect to the benzamine ring has been shown to considerably reduce the emission properties of ThT.^40,49,50^ For a coplanar conformation of both rings, the dihedral angle may present values close to 0°. As observed along the MD simulations, ThT keeps both rings in the same plane with dihedral angle values below 50° with mean values around ±10°.

**Figure 2:**
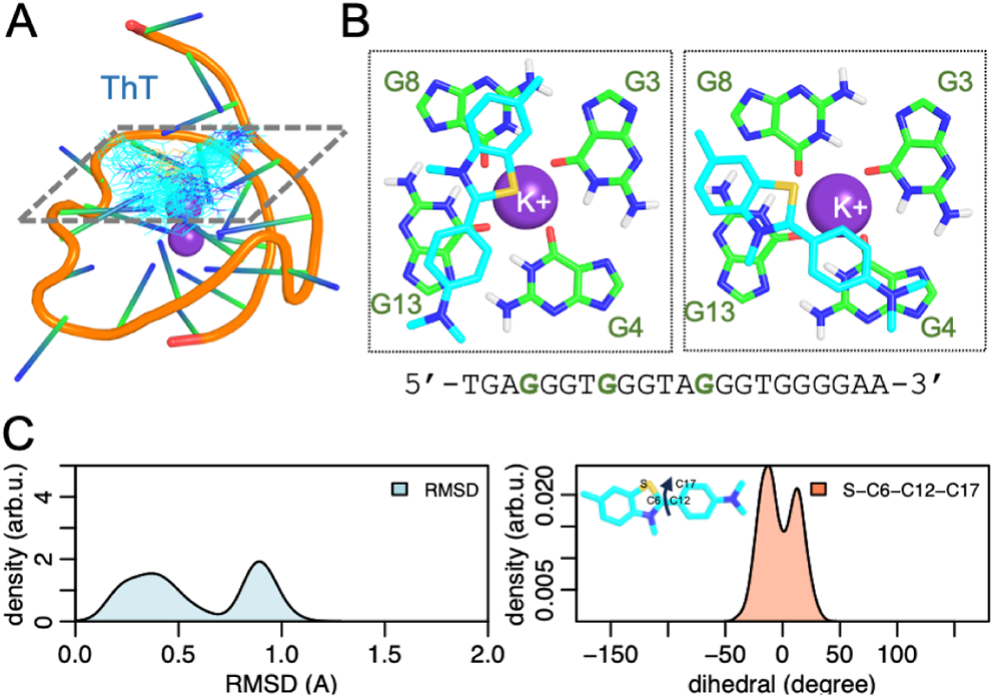
(A) Binding mode of ThT (shown as lines, C atoms in light blue) to *c-Myc* G4 (cartoons) along the MD simulation. 500 snapshots for ThT are superimposed. The interacting nucleobases are highlighted with dotted lines and marked in bold green in the *c-Myc* sequence. (B) Upper view of the plane of interaction between ThT (shown as sticks, C atoms color in blue) and the four guanine nucleobases in *c-Myc* G4 (shown as sticks, C atoms colored in green). (C) RMSD (Å) distribution along the MD simulation (1.5 μs) and distribution of the dihedral angle (degree) defined by S–C6–C12–C17 in ThT.

### Emission Event of ThT Bound to *c-Myc* G4

ThT has a molecular rotor nature:^51^ the rotation around the bond connecting the ThT benzothiazole and aminobenzene rings influences the quantum yield for the emission event. If both rings are perpendicular, the emission process is turned off. Thus, binding to macromolecules like amyloid fibrils or DNA increases the emission intensity of ThT when compared to aqueous solution due to rotational constraints.^51^ Based on these findings, we decided to study the emission process of ThT when bound to *c-Myc* G4 as a proof-of-concept of the binding mode obtained via MD simulations. For this purpose, we ran quantum mechanics/molecular mechanics (QM/MM) MD simulations of ThT bound to G4 (see Experimental Section). We used the oscillator strength (*f*_osc_) as an estimation of the fluorescence intensity (Figure 3). In our QM/MM simulations, we observed a significant decrease in emission intensity when ThT is in water compared with its bound form (Figure 3A). Nevertheless, we did not see a total loss of the emission in water. Looking at the former dihedral angle S–C6–C12–C17, we observed a broader range for this dihedral angle along the QM/MM simulation in water, but very few conformations visited the perpendicular disposition of both rings (Figure 3B). Nevertheless, we observed a larger charge transfer character (CT) in solution compared to that of the G4-bound form (Figure 3C). The population of low-lying energy dark states, with larger CT character compared to emissive states, prevents the relaxation of the excited electron via photon emission. Therefore, the emission intensity is lower in the case of ThT in solution.

**Figure 3:**
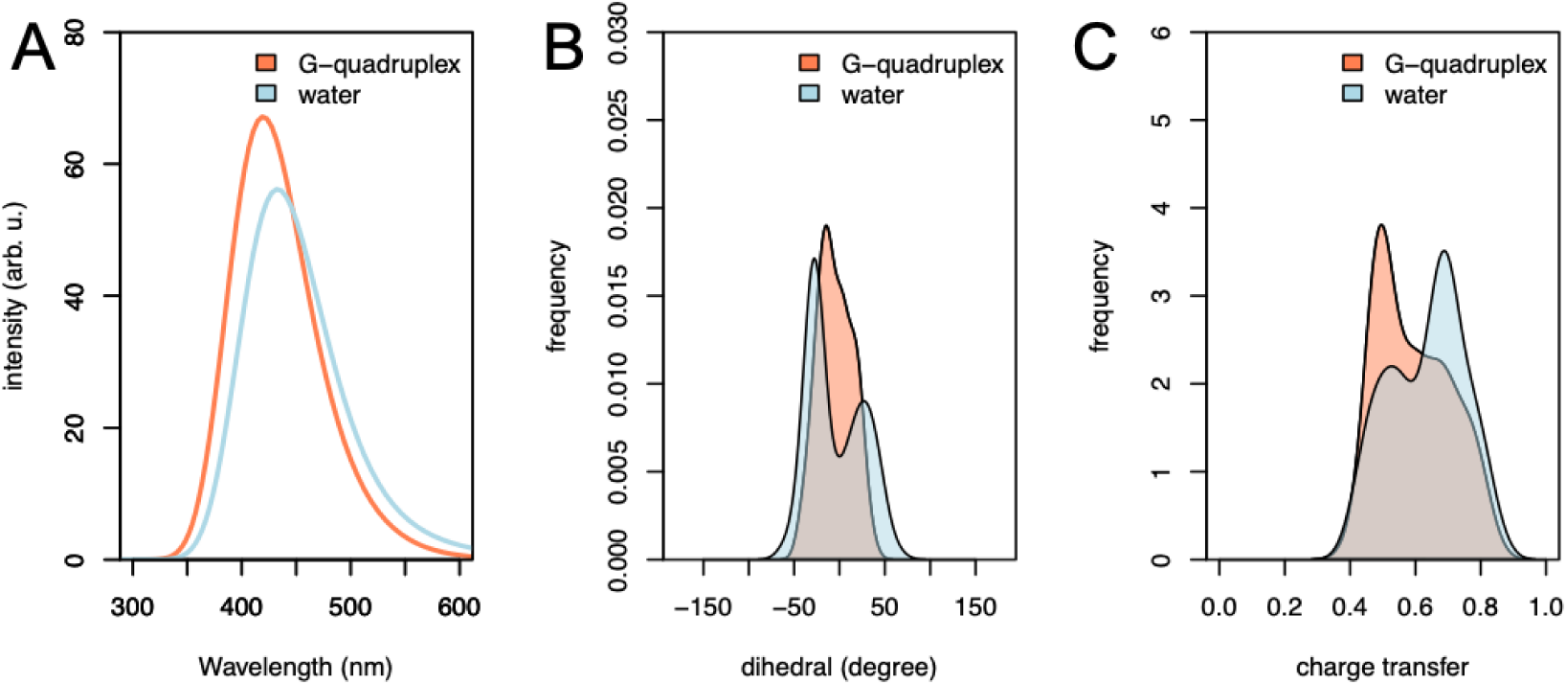
(A) Computer emission spectrum of ThT bound to *c-Myc* G4 (coral curve) and in solution (light blue). (B) Distribution of the dihedral angle (degree) defined by S–C6–C12–C17 in ThT bound to DNA (coral) and in water (light blue). (C) Charge transfer character for the electronic transitions during the emission process.

### Binding Mode of Natural Ligands to c*-Myc* G4

Next, we modeled the binding of the five natural products we tested to *c-Myc* G4 following the same protocol as that for ThT (Figure 4A). As a reference, we included the high-affinity synthetic binder TMPyP4. All compounds remain bound to G4, as shown by the distribution of RMSD for the ligands along the MD simulations (Figure 4B): all of them exhibit RMSD values close to 1 Å. Interestingly, EC and EGCG seem to have more fluctuations in the RMSD values than the rest of the compounds. Compared to the ligands, the G4 shows more flexibility during the MD simulations. We further computed the binding energies of each of the compounds to *c-Myc* G4 (Figure 4C). We used a classical force field provided by MM-ISMSA.^52^ By and large, the synthetic binder TMPyP4 shows a more negative binding energy, which agrees with the experimental results. The natural ligands show a binding energy in the range of ThT. Among the natural ligands, EGCG is the one with the largest binding energy in our calculations, and GA has the least negative binding energy. Brucine shows a moderate binding energy in our calculations.

**Figure 4:**
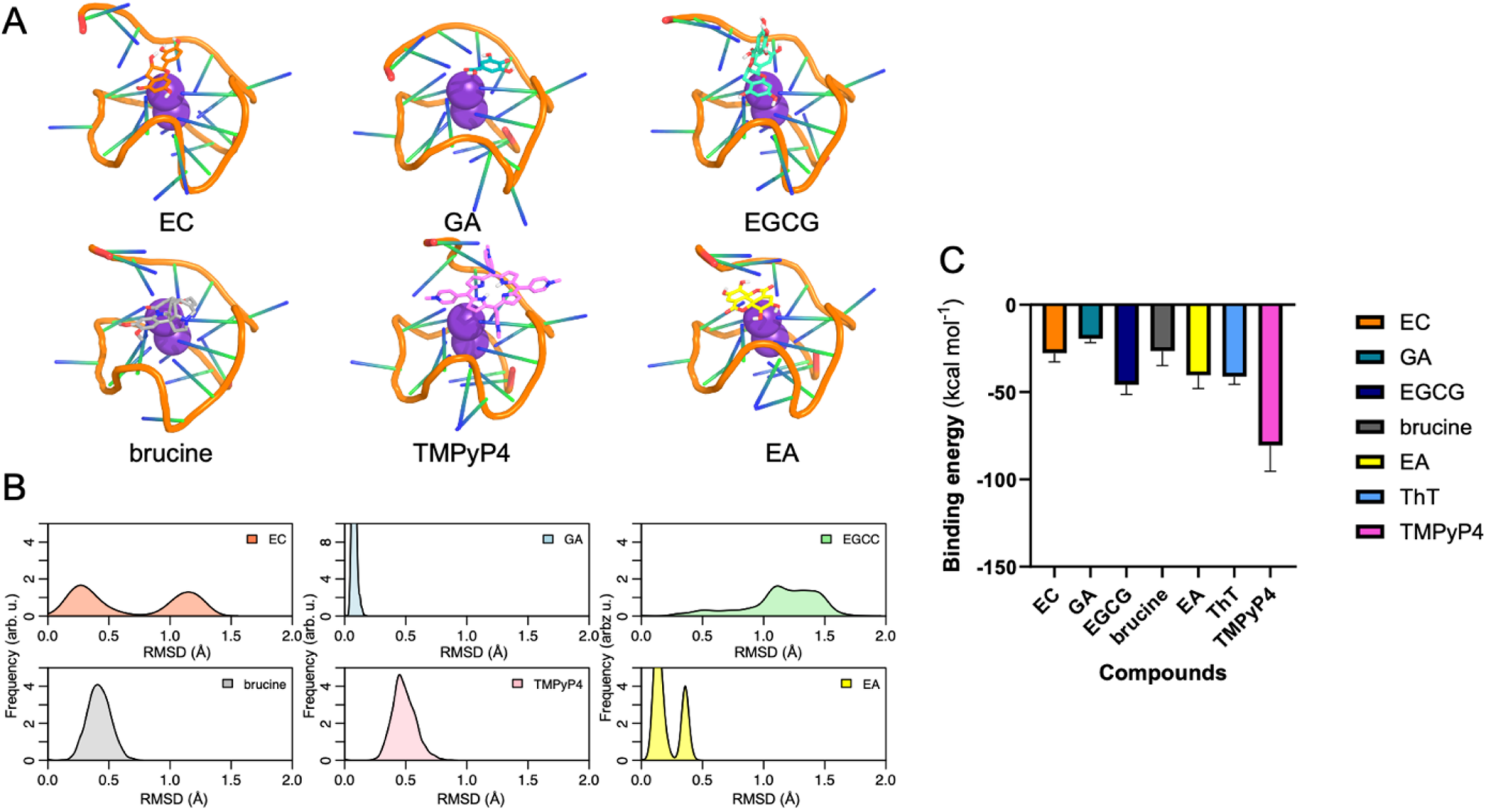
(A) Representative structure of the bound forms of the natural ligands to *c-Myc* parallel to G4. (B) Root-mean-square deviation (RMSD, Å) of the heavy atoms of the natural ligands, the probe ThT, and the synthetic ligand TMPyP4 along the MD simulations. (C) Binding energies (kcal mol^–1^) calculated along the MD simulations with MM-ISMSA.

To gain further insight into the chemical features that govern the binding of these ligands to G4s, we also analyzed intermolecular noncovalent interactions in the complexes (Figure 5). Compared to the previous binding energies calculated classically, the NCI analysis reveals that weak noncovalent interactions (green/light blue iso-surfaces) dominate the binding of all six ligands to the *c-Myc* G4, primarily through π–π stacking interactions of the aromatic rings and, additionally, CH−π, CH–O, and CH–N interactions. Some ligands also display classical hydrogen bonds (blue discs, Figure 5). In particular, EC, GA, and EGCG form hydrogen bonds through their NH and OH groups with the oxygen atoms of the nucleobases and phosphate backbone. In contrast, TMPyP4 lacks classical hydrogen bonds and relies on CH–O-like interactions. Brucine also establishes one NH–O hydrogen bond, which is further stabilized by a weaker CH–N interaction with the same nucleotide, highlighting the role of cooperativity between these two interactions. In this regard, EA shows the most pronounced cooperativity, forming two classical hydrogen bonds (NH–O and OH–N) with one nucleobase and an OH–O bond with a phosphate group.

**Figure 5:**
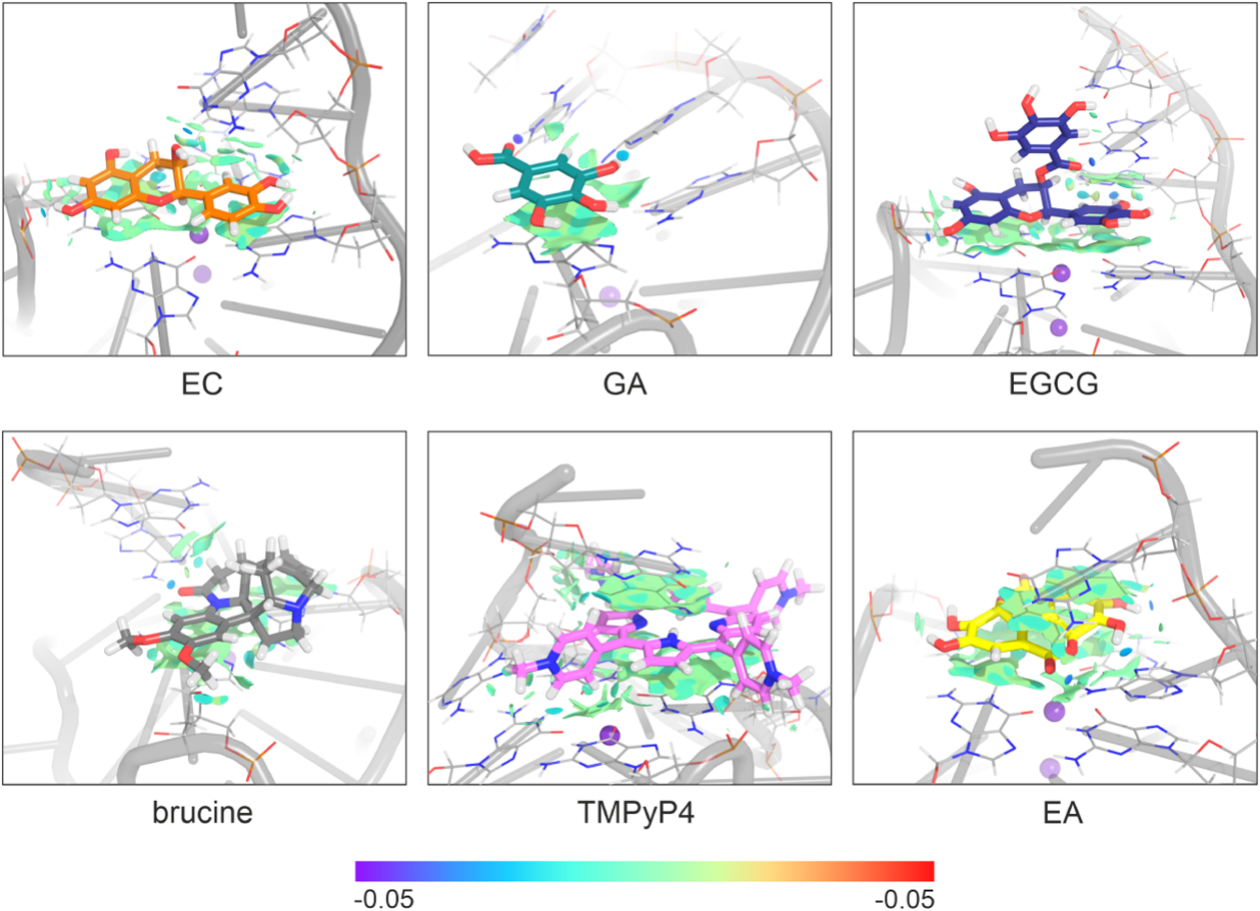
Noncovalent interactions between ligand (shown as sticks) and G4s (gray cartoon). The iso-surfaces report for the interactions represent the reduced density gradient (RDG), from strong interaction (blue iso-surface) to strong repulsion (red iso-surface), including very weak interactions (green iso-surface). Blue disks refer to hydrogen bonds.

We also evaluated the effect of the binding of the natural ligands on the stability of the G4-forming sequence of *c-Myc* during the MD simulation. We analyzed the MD trajectories based on the spatial coordinates of the DNA atoms and the root-mean-square deviation (RMSD) values for all atoms with respect to the initial conformation of the DNA G4. To reduce the dimensionality of the analysis, we used time-lagged independent component analysis.^53^ As an outcome, we obtained a statistical energy map of all conformers with respect to the first two independent components (ICs) of the TICA analysis (Figure 6). The projection with respect to IC 1 and IC 2 shows that the DNA visits different minima during the MD simulations. Although all the ligands bind to the same binding site, we found that the G4 in its bound form does not visit the same conformational states in all cases. As an example, ThT visits all minima shown in the statistical energy surface. A similar behavior is observed for the synthetic binder TMPyP4, although it covers different regions of the surface. In contrast, the rest of the ligands do not cover all minima. Brucine has a profile similar to ThT, whereas EA populates regions visited by TMPyP4. An intermediate region is visited by epicatechin, gallic acid, and EGCG.

**Figure 6:**
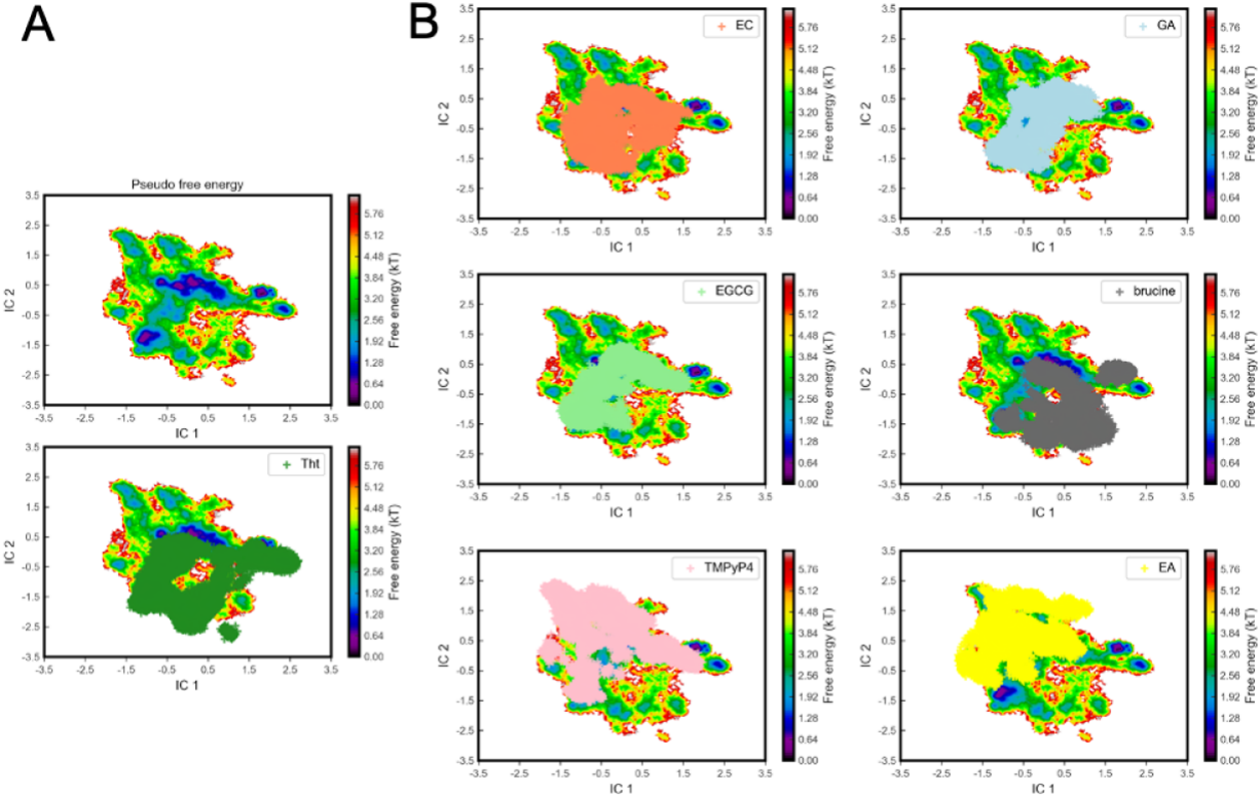
(A) G4 conformer distribution on the two internal coordinates (ICs) after TICA analysis for all systems and for ThT (green crosses) with respect to all states. The statistical energy (kT) is shown for each of the conformers. The statistical energy distribution of ThT is shown at the bottom. (B) Statistical energy distribution of each ligand with respect to all states.

We extended the analysis to principal component analysis (PCA) with features describing hydrogen bonds within the G-tetrads, the stacking of consecutive layers of G-tetrads, and the relative position of the ligand to the G4 guanines (Figure S3). This way, we can capture the changes in the G4 structure and the binding pose of the ligands. The projected data show two clear distributions for all ligands. Whereas ligands like brucine or EA explore the same region as the synthetic binder TMPyP4 and ThT, ligands like EGCG and GA populate another landscape.

### Cytotoxicity Tests

The cytotoxicity of all selected natural compounds was tested on MCF-7 cells by using the MTT viability assay (Figure 7). The commonly used cytostatic drug doxorubicin was used as a positive control for cytotoxicity. The results show that even at the highest concentration applied, 125 μM, none of the selected natural products have sufficient cytotoxic effects to reach the IC50 value. In contrast, the positive control reaches this value at a concentration of 2.5 μM. On the other hand, some of the compounds have been observed to enhance cell viability, for example: gallic acid, ellagic acid, or brucine. Based on these tests, we can assume that these compounds are safe to use in a potential treatment at these concentrations.

**Figure 7:**
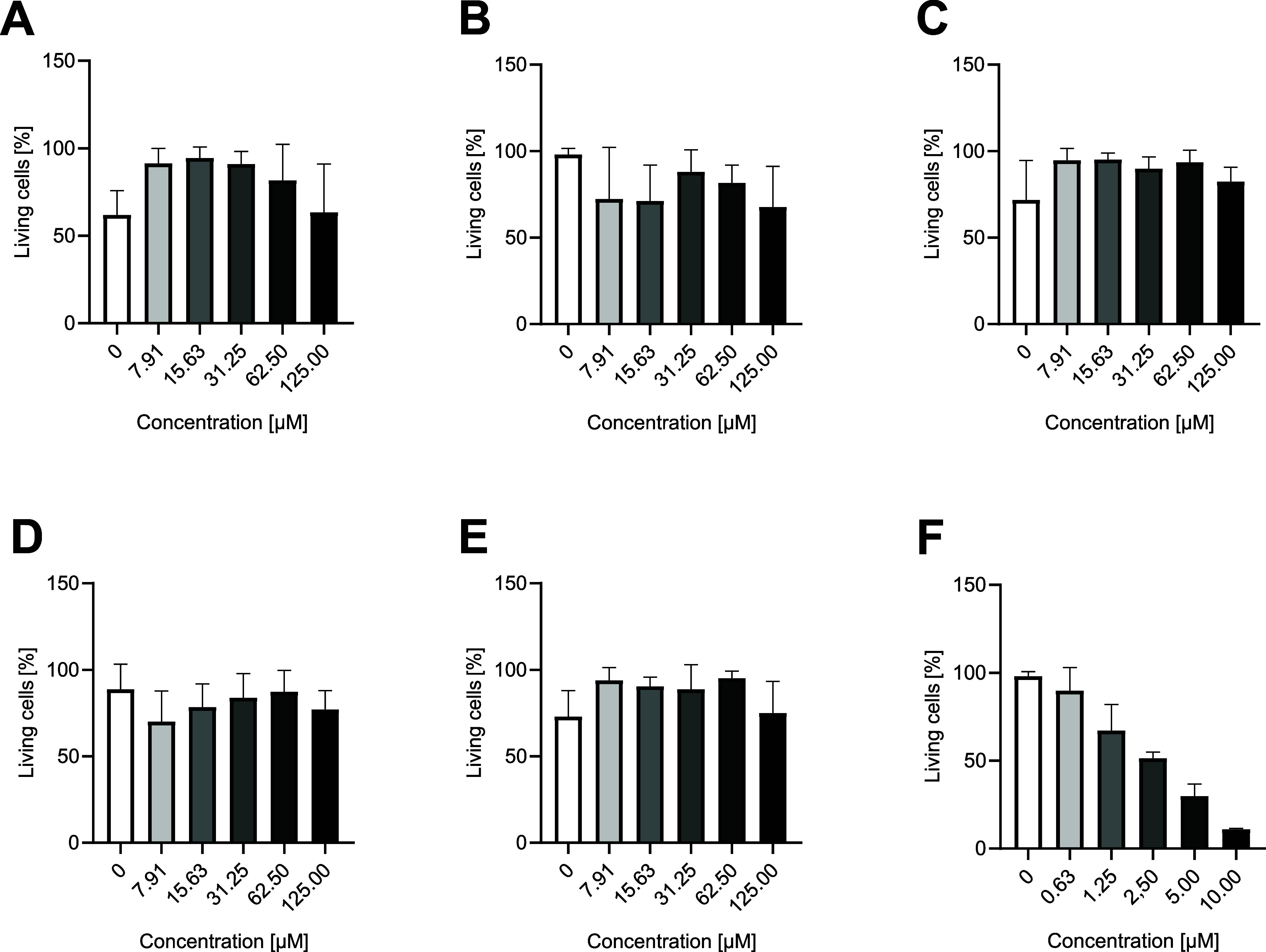
Viability assay (MCF7-cell line): (A) gallic acid, (B) EGCG, (C) ellagic acid, (D) epicatechin, (E) brucine, and (F) doxorubicin. The *x*-axis represents the concentration, and the *y*-axis represents the % of living cells, where 100% represents cells without treatment. Each experiment was repeated 3 times in triplicate; the results are presented as the mean, and error bars represent ± the standard deviation.

### Binding of Natural Ligands to G-Quadruplexes

Finally, we measured experimentally the ability of the five selected compounds to bind to the G4s. We used the thioflavin T (ThT) competition assay to test the ability of natural compounds to interact with the G4 (Figure 8). The propensity of tested sequences to form G4s was evaluated by CD (Figure S2). Whereas *c-Myc* forms a parallel G4, hTel structures as a hybrid G4. In its bound form, ThT emits light in the green range (λ_max_ = 490 nm). As soon as ThT is in solution, the intensity of the emission drops considerably (turn-off effect of the fluorescence). Therefore, the decrease in the fluorescence intensity of the tested compounds can be taken as an indication of the binding potency of these compounds: the level of signal reduction correlates directly with the amount of bound substance. In Figure 8, the decrease in fluorescence signal with increasing ligand concentration is evident for both hTel (Figure 8A) and *c-Myc* (Figure 8B) sequences, indicating that these natural substances expel ThT into the solvent and, most likely, occupy the ThT binding site. At low concentrations (<50 μM), EA and EGCG show the largest ThT-displacement degree of the tested compounds. At higher concentrations, brucine shows a larger reduction in the fluorescence signal. Brucine reaches baseline values at 125 μM with an approximately 80% reduction in fluorescence (Figure 8C). In contrast, the fluorescence assay results for EA show that the natural ligand displaces ThT not as effectively or strongly as brucine. The partial fluorescence reduction at higher concentrations suggests that EA may have a moderate binding affinity to the G4 DNA. At the highest used concentration, some ligands reached the DC_50_ value, the concentration at which 50% of the originally bound ThT was displaced. For the *c-Myc* oligonucleotide, this was achieved by EGCG, while for the hTel sequence, gallic acid, EGCG, and ellagic acid reached this threshold. In contrast, some compounds did not reach the DC_50_ value, even at the highest concentration tested. For both sequences, epicatechin was present; additionally, gallic acid did not reach DC_50_ in the case of *c-Myc*. The most effective compound for both sequences was brucine, which exceeded the DC_50_ threshold. The calculated DC_50_ values for brucine were 67.36 μM for the *c-Myc* oligonucleotide and 57.05 μM for hTel. Overall, these findings suggest that brucine has a higher binding affinity for the *c-Myc* G4 structure compared to that of EA, EGCG, GA, or epicatechin. All tested natural compounds were able to compete with the statistical significance of ThT for G4 binding at higher concentrations. As a control, we tested the natural compounds also for binding to hTelC, which is the former hTel sequence where the guanine nucleobases have been changed to cytosine. All the emission intensities are 1 order of magnitude smaller than for *c-Myc* or hTel and do not change with the concentration of the ligand.

**Figure 8:**
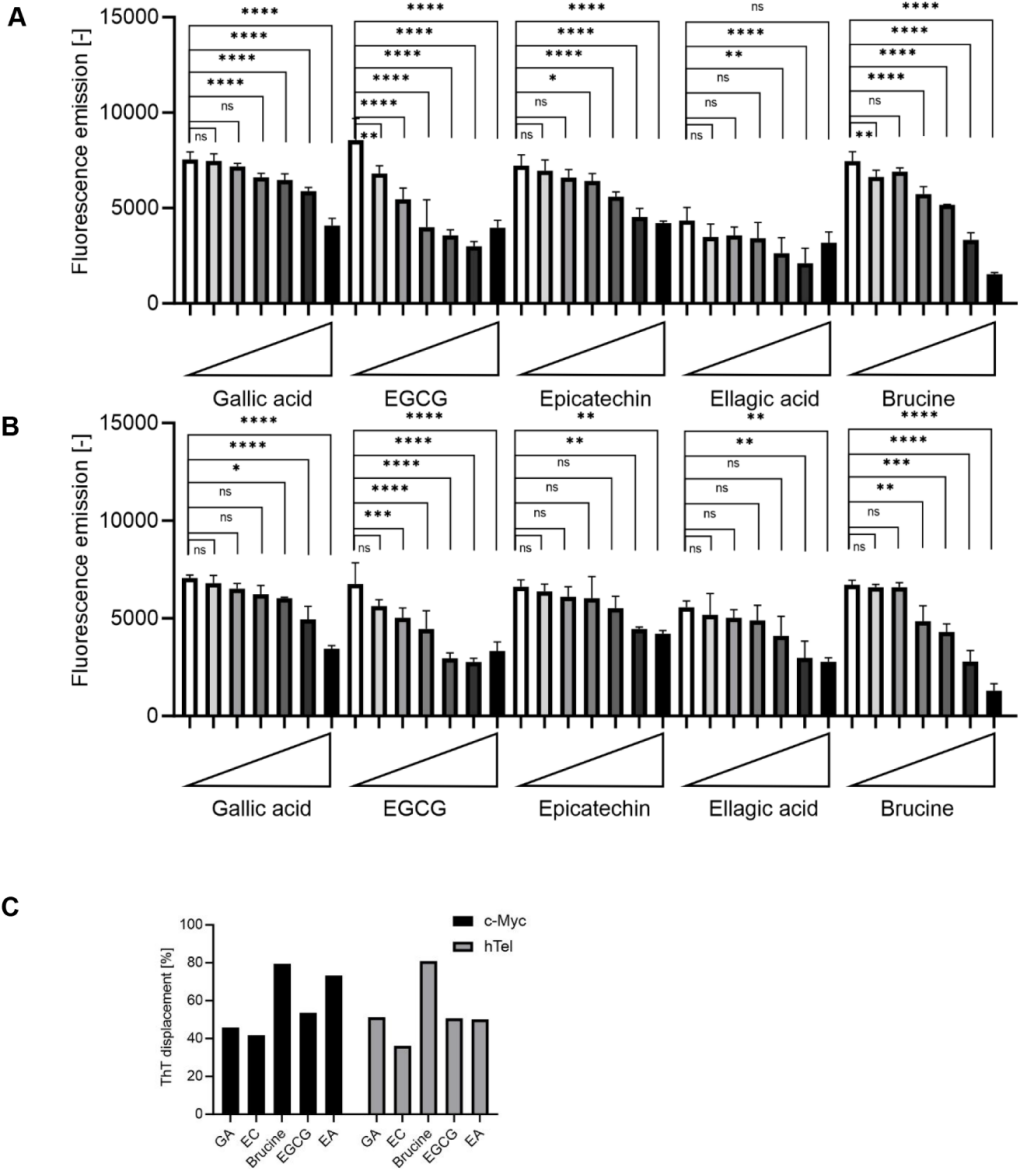
ThT displacement assay: (A) hTel sequence; (B) *c-Myc* sequence; (C) the percentage difference between the fluorescence intensity of *c-Myc* and hTel oligonucleotides with ThT itself without and with the addition of the ligands at the highest used concentration (125 μM). This difference is referred to as ThT displacement. Data are presented as mean ± standard deviation (SD) of at least nine biological replicates. The symbols * and **** indicate differences with *p* < 0.05 and *p* < 0.0001, respectively, between oligonucleotides without a natural ligand and with a natural ligand in different concentrations, and ns stands for not significant. The statistical test was done by a one-way ANOVA test followed by Tukey’s test.

### Discussion and Conclusions

Targeting DNA G4 structures with small molecules, including natural compounds, is a relevant strategy for the development of new therapeutic agents in cancer and other diseases.^38,54,55^ Although several G4 ligands have already entered clinical trials, these substances need to be further studied for their antitumor activity based on their targeting of oncogenes and tumor suppressors, which has been demonstrated in preclinical studies.^56–58^ Moreover, G4s are associated with the human genes involved in oncogenesis.^59–61^ Thus, the understanding of the chemical nature of the interactions between ligands and the DNA G4 can help in the design of more potent binders.^62–64^ In this work, we have selected five natural compounds, which have been shown to have the ability to bind to different structures of DNA:^38,65,66^ brucine, GA, EA, EC, and EGCG, to test their ability to bind to human *c-Myc* in silico, and for in vitro experiments, we also included human telomere G4-forming sequence.

The extensive MD simulations of the different ligands in complex with *c-Myc* G4 shed light on the chemical nature of the interaction. On the one hand, ThT stacks simultaneously with two guanine bases of the G4, with the sulfur atom pointing to the electron-deficient potassium ion. On the other hand, the binding of the natural ligands is based on weak interactions, mainly via π–π stacking interactions with the G4, although some ligands show additional hydrogen bonds. The superior binding capacity of brucine and, to a lesser extent, EA among the studied ligands, can then be attributed to cooperative hydrogen bonding, which stabilizes the interaction beyond individual contributions. These results suggest that cooperative hydrogen bonds can play a pivotal role in designing more potent G4 binders. In addition to that, since we do not include entropy in these calculations, we consider that the discrepancy between the strong ThT-displacement signal shown by brucine and the moderate binding potency obtained via MD simulations may be explained by other factors in addition to the binding enthalpy. We also looked at the binding of the tested compounds to *c-Myc* G4 via the TICA analysis. We show that the DNA structure adopts different conformers depending on the bound chemotype. To test the *in vitro* ability of natural ligands to bind to human *c-Myc* and hTel G4s, we used the ThT displacement assay, where there is a turn-off effect when ThT is displaced to the solvent upon the addition of the ligand of interest. Brucine, and to a lesser extent ellagic acid, interact strongly with both human *c-Myc* and hTel G4, and the rest of the compounds show a similar behavior at high μM concentrations.

Our data confirm that brucine can bind to both *c-Myc* and hTel G4 structures with micromolar affinity and that it is not toxic to the MCF7 cell line. The other tested natural compounds show weak affinities based on the ThT displacement assay. Computational studies reveal the cooperativity between π–π stacking and hydrogen bond interactions in the binding mode of brucine, and to a lesser extent in EA, may explain the superior binding potency compared to the other four ligands. Thus, the design of new compounds based on this scaffold may afford a versatile DNA G4 binder. However, the relatively high DC_50_ values observed for the tested plant-derived secondary metabolites suggest limited G4 stabilization under physiological conditions. Therefore, their direct therapeutic utility remains uncertain; nevertheless, these findings highlight potential starting points for the rational design and optimization of G4-targeting agents.

## Experimental Section

### Molecular Dynamics (MD) Simulations

The natural ligands gallic acid (GA), (−)-epigallocatechin gallate (EGCG), (−)-epicatechin (EC), ellagic acid (EA), and (−)-brucine were represented as GAFF atom types^67^ (Wang et al., 2004). The point charges were computed via the AM1-BCC model using Antechamber.^68^ The DNA was represented with the OL15 force field,^69^ and the TIP3P water molecule model was used for the solvent.^70^ The Cartesian coordinates of the parallel *c-Myc* G4 (3′-TGAGGGTGGGTAGGGTGGGGAA-5′) were obtained from the NMR-solved structure of this *c-Myc* promoter G4 in complex with berberine (PDB ID: 7N7D).^48^ The natural ligands were manually docked into DNA G4 by structural superimposition to berberine. All complexes were solvated with a box of 13 Å in size from the solute and neutralized with Na^+^. The solvated systems were then minimized in three steps, where hydrogens, solvent molecules (water and counterions), and the solute molecules were able to relax sequentially. The minimized system was heated up from 100 to 300 K in 20 ps using the Langevin thermostat (collision frequency of 1.0 ps^–1^) at constant volume and temperature (NVT ensemble). All solute atoms were restrained with a harmonic force of 200.0 kcal mol^–1^ Å^–2^. Before production, the system was equilibrated in five steps (265 ps). During this equilibration process, the system switched from constant volume to constant pressure (NPT ensemble) and the restraints were removed from the solute atoms. Each of the ligand/G4 complexes were simulated for a total time of 1.5 μs as three independent simulations (3 × 0.5 μs). The SHAKE algorithm^71^ was applied, and a time step of 2.0 fs was defined. All molecular dynamics (MD) simulations were conducted using the Amber20 software package.^72^ The root-mean-square deviation (RMSD, Å) values of each DNA: ligand complex as a function of time are summarized in Figure S1.

### Time-Lagged Independent Component Analysis (TICA) and PCA

To investigate the effect of ligand binding on the structure of DNA G4 during the MD simulations, we employed time-lagged independent component analysis (TICA) and Principal Component Analysis (PCA). TICA is a dimensionality reduction technique that identifies degrees of freedom with the slowest-relaxing time. We selected the following molecular features for TICA: the root-mean-squared deviation (RMSD, Å) of the DNA G4 in the presence of the ligand and the intramolecular distances (Å) in the G4 along the MD simulations (Figure 6). For the PCA, we selected the distances of the Hoogsteen hydrogen bonds formed in each of the G-tetrads, the distance between the geometrical center of each guanine present in a G-tetrad and the guanine in the G-tetrad immediately above, and the distance between the geometrical centers of the ligand and the geometrical center of each of the guanines present in the upper G-tetrad (Figure S3). In both cases, we projected all of the conformational states of the DNA G4, along the MD simulation using the first two independent components (IC_1_ and IC_2_ in TICA; PC_1_ and PC_2_ in PCA). Using the new dimensions, we calculated the total statistical free energy landscape (kT units) using histogram counting. A total of 105 000 frames were included in the analysis. All features and dimensionality reduction were done with the pyEMMA Python package.^53^

### Noncovalent Analysis of the Ligand–DNA Complexes

The noncovalent interactions between the ligands and the *c-Myc* G4 within 4.0 Å from the ligand were visually analyzed using the noncovalent interaction (NCI) index and the program NCIPLOT.^73,74^ For each complex, a representative structure from the major conformational cluster of the MD simulations was used for the NCI analysis. Using NCIPLOT, we represent the stabilizing interactions, van der Waals interactions, and repulsive interactions on the binding interface of the ligand/G4 as iso-surfaces. Green iso-surfaces denote van der Waals interactions; blue iso-surfaces indicate hydrogen-bonding or attractive forces; and red iso-surfaces indicate steric interactions.

### QM/MM MD Simulations and Computation of the Emission Spectra of ThT

Both emission spectra of ThT bound to *c-Myc* G4 and in solution were computed using conformational ensembles generated by means of QM/MM MD simulations. The ligand ThT was included in the QM region, and the rest of the system was treated classically and represented as point charges for the QM/MM partition scheme (electrostatic embedding). A total of 20 snapshots of the major conformational clusters from the MD simulations were randomly selected as the initial structures. Each of these systems was propagated in the ground-state S_0_ for 5 ps using xTB2/OL15 ^69,75^ with a time step of 1 fs. 100 snapshots were printed from the ground-state QM/MM-MD-based trajectories and further simulated with linear response Time-dependent DFT (LR-TD-DFT) using the Tamm–Dancoff Approximation (TD-DFT/TDA)^76^ for 500 fs in the first excited state S_1_ with a time step of 0.2 fs. The QM region was treated with Density Functional Theory (B3LYP), including the Grimme D3 dispersion correction with Becke–Johnson damping scheme^77,78^ together with a double-ζ basis set (def2-SVP)^79^ and the rest of the system treated classically. A Davidson expansion space of 5 states was defined. After that, static TD-DFT/TDA calculations using BP86 ^80^ with the same basis set were repeated for 100 snapshots from the former QM/MM-MD trajectory. The S_1_ energies and oscillator strength (*f*_osc_) were used to convolute the emission spectrum of ThT. Benchmark studies of various functionals have shown that in our case, the generalized-gradient approximation functional BP86 provides transition energies that are closer to the experimental emission spectrum. The emission spectra were convoluted with the program TheoDORE.^81^

### Natural Compounds

(−)-Brucine (CAS 357-57-3), (−)-epigallocatechin gallate (EGCG, CAS 989-51-5), (−)-epicatechin (EC, CAS 490-46-0), ThT as chloride salt (CAS 2390-54-7), and ellagic acid (EA, CAS 476-66-4) were obtained from Sigma-Aldrich. Gallic acid (GA, CAS 149-91-7) was obtained from Acros Organics (Thermo Scientific Chemicals). As a positive control, the tetratosylate salt of synthetic ligand TMPyP4 (CAS No. 36951-72-1, Sigma-Aldrich), known for its ability to bind G4, was selected.

### DNA

A set of three commercially synthesized oligonucleotides was obtained from Eurofins. The first sequence is the G4-forming sequence from the *c-Myc* promoter region (5′-AGCTTGAGGGTGGAGGTGGGAAA-3′). The second sequence is from the human telomere end region (hTel) (5′-AGCTTGGGTTAGGGTTAGGGTTAA-3′), and, as a negative control, a modified hTel sequence in which all guanines have been replaced by cytosines (hTelC) (5′-AGCTTTAACCCTAACCCTAACCCA-3′) was used.

### Cytotoxicity Tests

In order to utilize natural substances as potential treatments, it is imperative to ascertain their safety. To evaluate the suitability of these substances for humans, MTT (3-(4,5-dimethylthiazol-2-yl)-2,5-diphenyltetrazolium bromide) cytotoxicity assays were performed to assess their toxicity. In this assay, MCF-7 (a human breast cancer cell line) cells were exposed to natural compounds at concentrations ranging from 0 to 125 μM for 24 h. The yellow tetrazolium dye MTT was then added. MTT can be metabolically converted to insoluble purple formazan by enzymes in living cells. After 3 h of incubation, the formazan crystals were resuspended in a 500:1 solution of isopropanol and HCl, and the absorbance of each sample was measured spectrophotometrically at a wavelength of 590 nm.

### Fluorescence Displacement Assay

To experimentally evaluate the affinity of natural compounds for the G4 structure, thioflavin T (ThT) was used in the fluorescence displacement assay. Stock solutions were prepared in a buffer containing 100 mM Tris-HCl and 100 mM KCl, adjusted to pH 7.0. Before experimentation, oligonucleotide solutions underwent an annealing process involving heating to 95 °C for 5 min, followed by gradual cooling to room temperature within the same buffer system. The annealed oligonucleotides were titrated into black 384-well microplates (Corning) at room temperature, followed by the addition of the test molecules to each well, resulting in final concentrations ranging from 0 to 125 μM. Two lower concentrations (0.13 and 0.25 μM) were added for synthetic TMPyP4 due to its high ability to replace ThT; for the same reason, TMPyP4 is presented in a separate graph. ThT was then added to the mixture. The oligonucleotide and ThT were combined at a 2:1 molar ratio in a total volume of 20 μL, yielding final concentrations of 1 μM for the oligonucleotides and 0.5 μM for ThT. Fluorescence emission at 490 nm was captured after excitation at 425 nm using a Spark fluorescence microplate reader (Tecan). All measurements were performed at room temperature, with triplicate readings taken for each concentration of the test molecule, as well as for the negative control, which consisted of an oligonucleotide in buffer with ThT alone.

### Circular Dichroism (CD) Spectroscopy

Stock solutions were prepared at 2.2 μM in 100 mM Tris-HCl buffer with 100 mM KCl, pH 7.5. The oligonucleotide solutions were annealed by heating at 99 °C for 5 min, then slowly cooled to room temperature. Circular dichroism (CD) measurements were taken by using a Jasco 815 dichrograph (Tokyo, Japan) in 1 cm quartz Hellma cells at 23 °C. CD spectra were collected from 230 to 330 nm, with a 0.5 nm data pitch, 100 nm min^–1^ scan rate, and four accumulations. The CD signal was expressed as the molar absorption difference, Δε [M^–1^ cm^–1^], between left- and right-handed circularly polarized light, with molarity based on DNA strands.

### Statistical Evaluation

All experimental data were averaged from a minimum of 8 values for the cytotoxicity assays and from a minimum of 9 values for the ThT assays, and the standard deviation (SD) was determined for each average. For the ThT displacement assay test results, the ANOVA statistical test was used to determine the significant difference between the resulting values, and the Tukey’s test was used to determine the significance level. The symbols for significance * and **** indicate differences with *p* < 0.05 and *p* < 0.0001.

## Highlights

- Plant-derived secondary metabolites brucine, gallic acid, ellagic acid, epicatechin, and epigallocatechin gallate were tested for G-quadruplex binding properties.
- Brucine exhibits the strongest interaction with G-quadruplex structures.
- π–π stacking and H-bonding contribute to the high binding affinity of tested compounds.
- Displacement of ThT into solution enhances charge transfer character in the excited states involved in ThT fluorescence.

## Supplementary Material



## Data Availability

The computational data have been deposited at 10.5281/zenodo.14288852.

## Notes

Root-mean-squared deviation (RMSD, Å) along the MD simulations for each of the elements of the simulated systems; the circular dichroism spectra of hTel and *c-Myc* DNA G4s; free energy landscape projected onto the first two components of the PCA for all ligands (PDF)

## Abbreviations

EGCG: (−)-epigallocatechin gallate
GA: gallic acid
EC: (−)-epicatechin
EA: ellagic acid
ThT: thioflavin T
B-DNA: right-handed double helix DNA
