# Supplementary material for: Study on the Binding of Five Plant-Derived Secondary Metabolites to G‑Quadruplexes
Source: ACS Omega. 2026 Jan 5;11(2):3096–107. doi: 10.1021/acsomega.5c09032 (PMC12824740; doi:10.1021/acsomega.5c09032)
Supplement: Supplementary file 1 [file ao5c09032_si_001.pdf]

Supporting Information

Study on the Binding of Five Plant-Derived Secondary Metabolites to G-Quadruplexes

*Lucie Vrtalova[1,2], Michaela Dobrovolná[1,2], Daniel Platero-Rochart,[3] Aleksandra L. Ptaszek,[3,4] Václav Brázda[1,*2], Pedro A. Sánchez-Murcia[3,5*]*

[1]Institute of Biophysics of the Czech Academy of Sciences, Královopolská 135, 612 00 Brno, Czech Republic
[2]Brno University of Technology, Faculty of Chemistry, Purkynova 118, 612 00 Brno, Czech Republic
[3]Laboratory of Computer-Aided Molecular Design, Division of Medicinal Chemistry, Otto-Loewi Research Center, Medical University of Graz, Neue Stiftingtalstrasse 6/III, 8010 Graz, Austria
[4]Christian Doppler Laboratory for High-Content Structural Biology and Biotechnology, Department of Structural and Computational Biology, Max Perutz Labs, University of Vienna, Campus Vienna Biocenter 5, 1030 Vienna, Austria
[5]BioTechMed-Graz, Mozartgasse 12/II, 8010 Graz, Austria

Abbreviations: EGCG: (-)-epigallocatechin gallate; GA: gallic acid; EC: (-)-epicatechin; EA: ellagic acid; ThT: thioflavin T; B-DNA: right-handed double helix DNA.

*Corresponding author: vaclav@ibp.cz (V Brázda); pedro.murcia@medunigraz.at (P.A. Sánchez-Murcia)

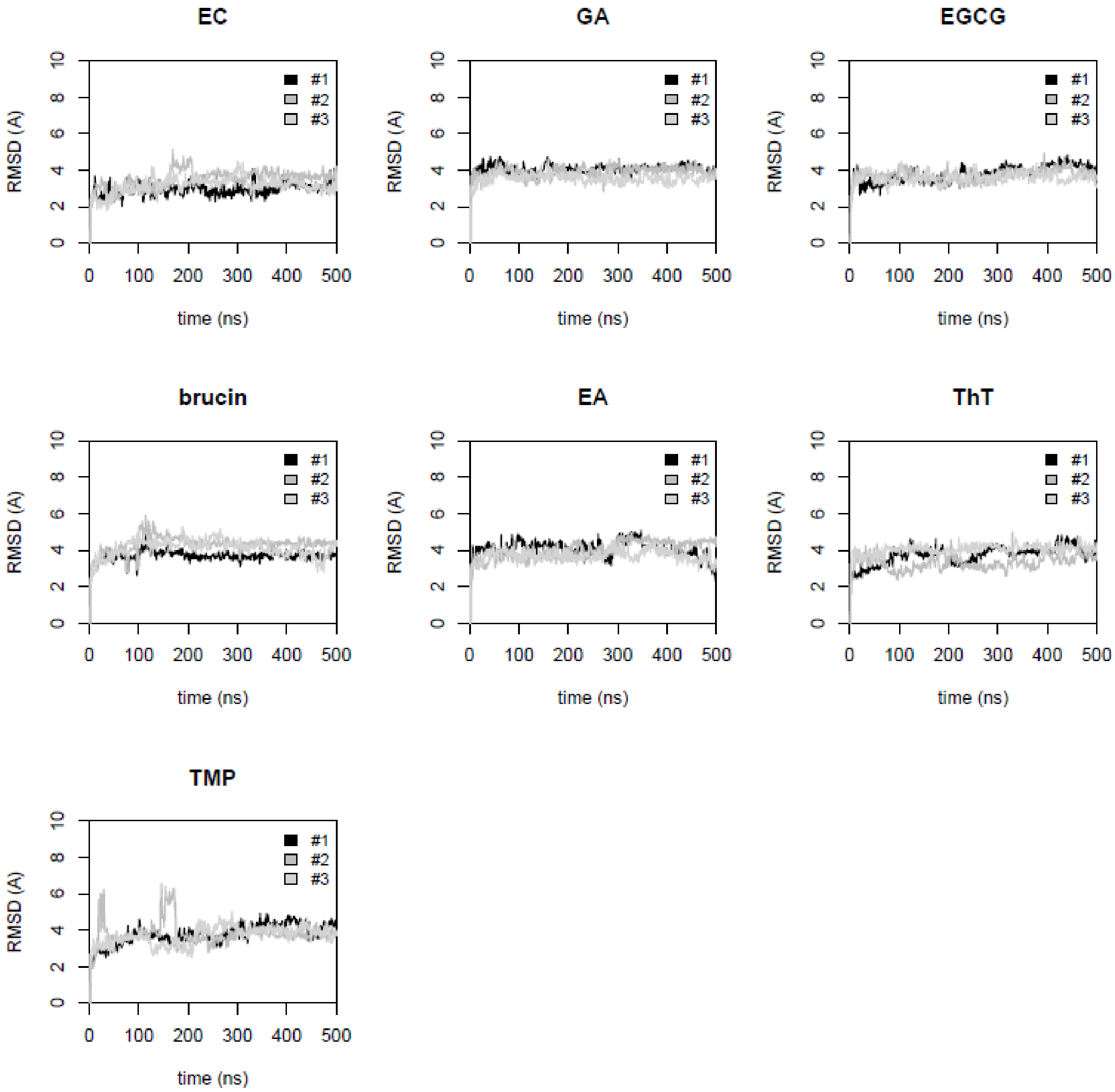

Figure S1. Root-mean square deviation (RMSD, Å) of G-quadruplex and ligands along the classical MD simulations.

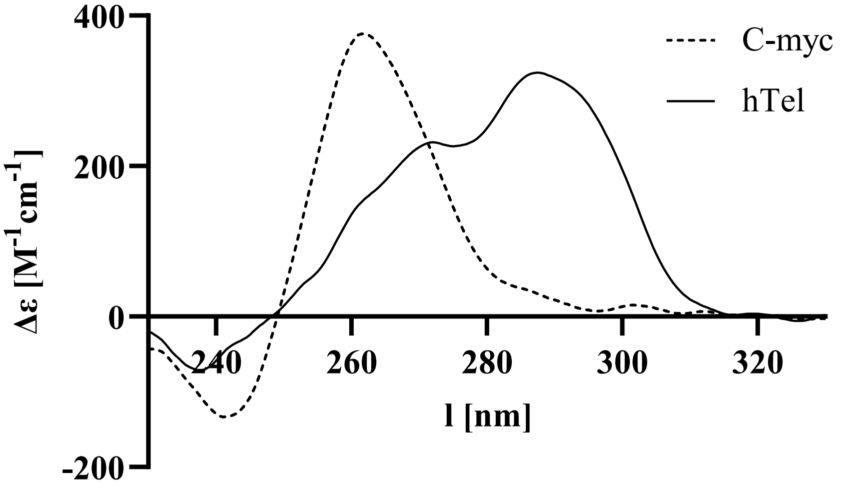

Figure S2. Circular dichroism (CD) spectra comparing hTel (solid line) and c-Myc (dashed line) oligonucleotides. The distinct CD profiles highlight differences in their secondary structures, with c-Myc showing characteristic signals indicative of parallel G-quadruplex formation, while the hTel sequence shows prominent peaks typical of a hybrid G-quadruplex structure.

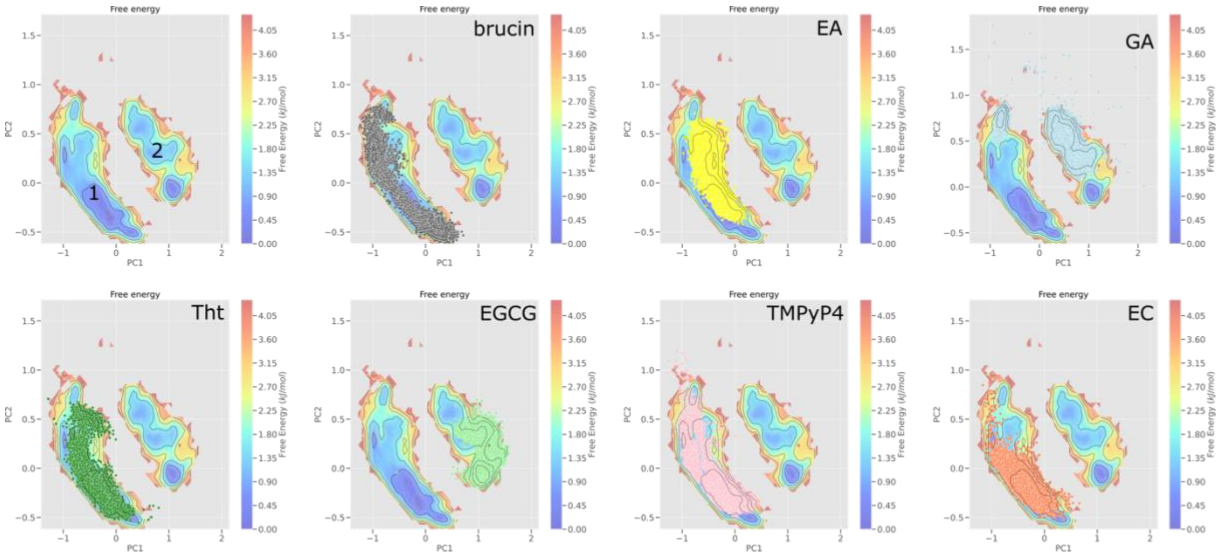

Figure S3. Free energy landscape projected onto the first two components of the PCA (PC1 and PC2). The region explored by each simulation is represented in scatter points. The plots clearly show the difference in the explored regions by probe Tht, the synthetic ligand TMPyP4, brucin, EC and the ligands GA, and EGCG.
